# Alcohol Use and Risk of Dementia in Diverse Populations

**DOI:** 10.1136/bmjebm-2025-113913

**Published:** 2026-01-21

**Authors:** Anya Topiwala, Daniel F. Levey, Hang Zhou, Joseph D. Deak, Keyrun Adhikari, Klaus P. Ebmeier, Steven Bell, Stephen Burgess, Thomas E. Nichols, VA Million Veteran Program, J. Michael Gaziano, Murray B Stein, Joel Gelernter

**Affiliations:** Nuffield Department of Population Health, Big Data Institute, University of Oxford, Oxford, UK, OX3 7LF; Department of Psychiatry, Yale University School of Medicine, New Haven, CT, USA; Veterans Affairs Connecticut Healthcare System, West Haven, CT, USA; Department of Psychiatry, Yale University School of Medicine, New Haven, CT, USA; Veterans Affairs Connecticut Healthcare System, West Haven, CT, USA; Section of Biomedical Informatics and Data Science, Yale University School of Medicine, New Haven, CT, USA; Department of Psychiatry, Yale University School of Medicine, New Haven, CT, USA; Veterans Affairs Connecticut Healthcare System, West Haven, CT, USA; Department of Psychiatry and Wellcome Centre for Integrative Neuroimaging, University of Oxford, UK; Department of Oncology, School of Clinical Medicine, University of Cambridge, Cambridge; Cancer Research UK Cambridge Centre, Li Ka Shing Centre, University of Cambridge, Cambridge, UK; MRC Biostatistics Unit, School of Clinical Medicine, University of Cambridge, Cambridge, UK; Department of Public Health and Primary Care, School of Clinical Medicine, University of Cambridge, Cambridge, UK; Nuffield Department Population Health, Big Data Institute, University of Oxford, Oxford, UK; Wellcome Centre for Integrative Neuroimaging, FMRIB, Nuffield Department of Clinical Neurosciences, University of Oxford, Oxford; Million Veteran Program, VA Boston Healthcare System, Boston, MA; Department of Medicine, Harvard Medical School, Boston, MA.; Department of Medicine, Divisions of Aging and Preventative Medicine, Brigham and Women’s Hospital, Boston, MA; https://www.nature.com/articles/s41593-024-01720-5; Department of Psychiatry and School of Public Health, University of California, San Diego, La Jolla, CA; VA San Diego Healthcare System, San Diego, CA; Genetics and Neuroscience, Department of Psychiatry, Yale University School of Medicine, New Haven, CT, USA; Veterans Affairs Connecticut Healthcare System, West Haven, CT, USA

## Abstract

**Objectives:**

To investigate the causal relationship between alcohol consumption and dementia.

**Design:**

Prospective cohort analysis combined with linear and nonlinear Mendelian randomization.

**Setting:**

Two large-scale population-based cohorts: the US Million Veteran Program and UK Biobank. Genetic analyses used summary statistics from genome-wide association studies (GWAS).

**Participants:**

559,559 adults aged 56–72 years at baseline were included in observational analyses (mean follow-up: 4 years in the US cohort; 12 years in the UK cohort). Genetic analyses used summary data from multiple large GWAS consortia (2.4 million participants).

**Main outcome measures:**

Incident all-cause dementia, determined through health record linkage, and genetic proxies.

**Results:**

During follow-up, 14,540 participants developed dementia and 48,034 died. Observational analyses revealed U-shaped associations between alcohol and dementia risk: higher risk was observed among non-drinkers, heavy drinkers (>40 drinks per week; hazard ratio [HR]=1.41, 95% confidence interval[CI] 1.15-1.74), and those with alcohol use disorder (AUD) (HR=1.51[CI 1.42-1.60]) compared with light drinkers. In contrast, Mendelian randomization analysis identified a monotonic increase in dementia risk with greater alcohol consumption. A two-fold increase in AUD prevalence was associated with a 16% increase in dementia risk (inverse-variance weighted [IVW] OR=1.16[1.03-1.30]). A one standard deviation increase in log-transformed drinks per week was associated with a 15% increase (IVW OR=1.15[1.03-1.27]). Furthermore, individuals who developed dementia experienced a greater decline in alcohol intake over time, suggesting reverse causation—where early cognitive decline leads to reduced alcohol consumption—underlies the protective effects observed in observational studies.

**Conclusions:**

These findings provide evidence for a causal relationship between alcohol use and increased dementia risk. While observational data suggested a protective effect of light drinking, genetic analyses did not support this, indicating that any level of alcohol consumption may contribute to dementia risk. Public health strategies that reduce the prevalence of alcohol use disorder could potentially lower the incidence of dementia by up to 16%.

## Introduction

Alcohol consumption is widespread, modifiable, and associated with many medical harms, but its causal relationship with dementia remains contentious. While heavy drinking has been associated with increased dementia risk in some cohorts, findings are inconsistent across different studies.^1^ The situation is even more ambiguous among moderate drinkers, with some research purporting protective effects of moderate alcohol consumption.^2^ However, recent neuroimaging studies have uncovered adverse associations with dementia endophenotypes, even at low levels of alcohol consumption.^3^ These findings highlight the complexity of alcohol’s impact on cognitive health, and underscore the urgent need to clarify the true effects of alcohol on dementia risk. This is of critical importance for public health, as it can guide risk awareness, inform preventative strategies, and influence health guidelines for individuals.

It has been proposed that the effects of alcohol on brain health may be nonlinear, with an “optimal” dose for health greater than zero. However, methodological differences across studies may explain the contradictory findings in the existing literature.^4^ Most studies have had limited inclusion of heavy or dependent drinkers, which restricts their power to detect the full range of alcohol’s effects.^4^ Additionally, many studies have involved elderly participants, where cognitive decline may influence drinking patterns rather than the opposite – i.e., reverse causation.^5^ The inclusion of nondrinking reference groups likely consisted of former heavy drinkers who are now abstinent,^6^ which introduces confounding (because such subjects are susceptible to long-term health effects of alcohol from prior heavy use), complicating efforts to draw definitive conclusions about causality. As a result, we are currently unable to confidently infer a causal relationship between alcohol consumption and cognitive decline.^7^ Practical and ethical issues preclude randomized controlled trials on alcohol use and dementia risk. However, Mendelian randomization (MR), a quasi-experimental approach leveraging genetic data, offers an opportunity to estimate causal effects.^8^ Five Mendelian randomization studies have investigated the linear relationship between alcohol consumption and late-onset Alzheimer’s disease in European ancestry-only populations, all reporting null findings,^9,10^ These studies were limited by statistical power, and in the context of alcohol’s broader impact on dementia, non-Alzheimer dementia etiologies may be more relevant.^11^ Moreover, previous studies, with one lower powered exception in White British individuals,^12^ did not examine the crucial question of nonlinear relationships with alcohol, precluded by relying on summary data from existing Alzheimer’s GWAS (genome-wide association studies). The absence of large-scale data on both alcohol use and diverse dementia phenotypes has hindered progress in answering a key question: Is there an optimal weekly alcohol intake for brain health? Additionally, if it is the case that even low or moderate alcohol intake is harmful to brain health, earlier research suggesting the contrary may have led individuals to intentionally increase alcohol intake to take advantage of its purported health benefits. More and better data are needed to optimize public health advice.

In this study, we aimed to estimate the role of alcohol use in dementia risk across the entire dose range, including in the moderate drinking range. To achieve this, we used two large and diverse biobanks: the Million Veteran Program (MVP)^13^ in the US and the UK Biobank (UKB).^14^ Given the current uncertainty about which specific subtypes of dementia alcohol could impact, we included a broad range of dementia phenotypes, rather than restricting our focus to Alzheimer’s disease, as has been done in prior studies. The primary data for these powerful analyses were generated through a novel biancestry GWAS of all-cause dementia in MVP, allowing for a crucial examination of nonlinear relationships between alcohol consumption and dementia risk at the largest scale to date. Our study also benefited from extensive longitudinal phenotype data, capturing how alcohol consumption patterns evolve in aging individuals, allowing us to explore the potential role of reverse causation between alcohol and dementia. To strengthen causal inference, we triangulated traditional observational methods with genetic analyses, exploring various alcohol-related traits across different genetic ancestries and examining how these factors interact with the aging process.

## Methods

### Study populations

**Figure 1:**
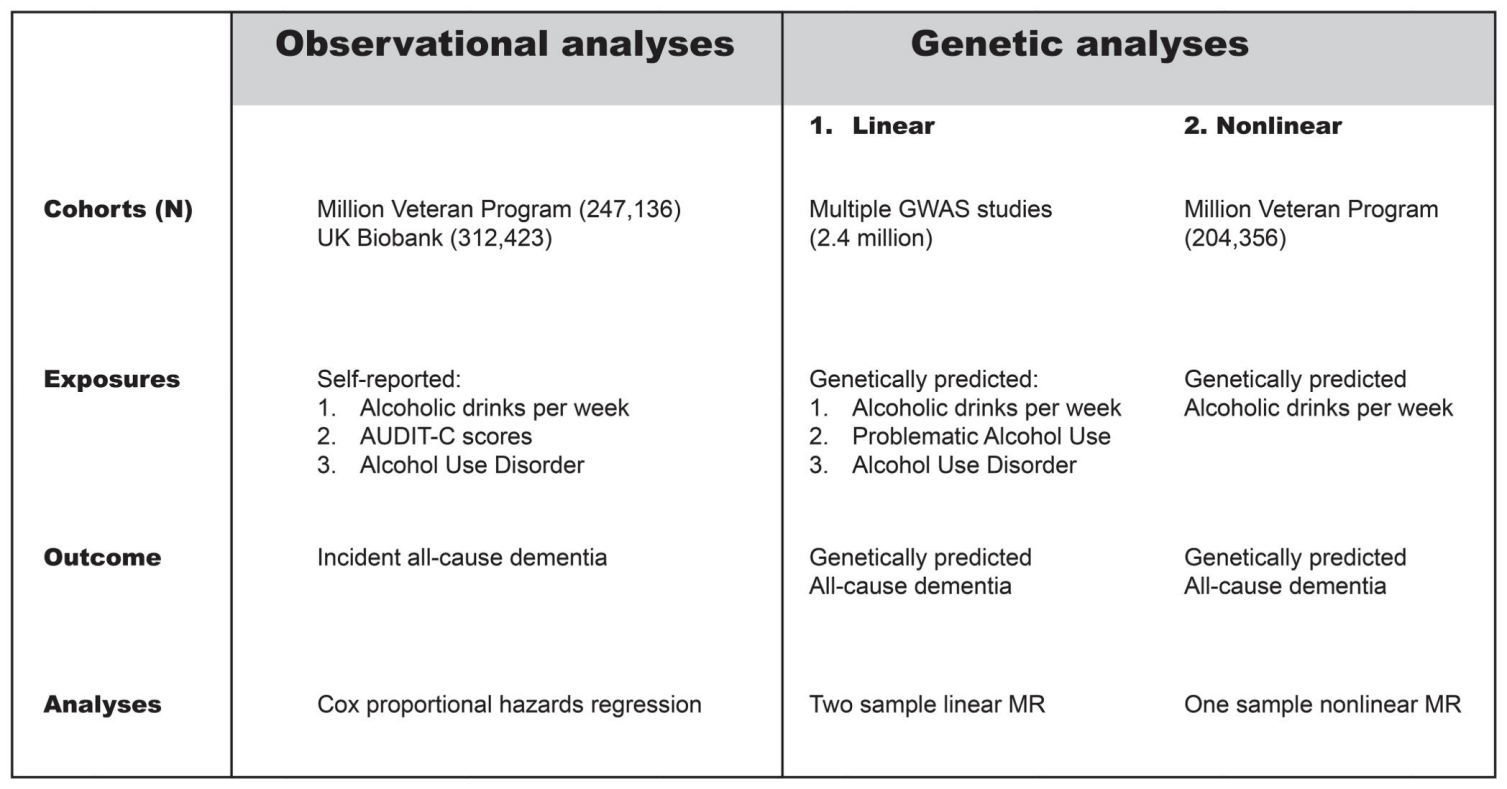
Analyses overview

The observational study utilized data from two large, diverse cohorts (Fig 1): the Million Veteran Program (MVP), and the UK Biobank (UKB). MVP includes US veterans who were recruited from 2011 to the present.^13^ UKB recruited volunteers aged 40-69 years from 2006 to 2010.^14^ Participants were followed up from recruitment until either their first dementia diagnosis, death, or the date of last follow up (December 2019 for MVP and January 2022 for UKB). Participants in both cohorts provided written informed consent, and the studies had approval from their respective institutional and ethics review boards. All individuals in analyses were unrelated and stratified by genetic ancestry to allow for analyses across diverse populations.

The genetic analyses included a total of 2.4 million participants from 45 genome-wide association study cohorts (Supplementary Table 18). These genetic cohorts facilitated the Mendelian randomization analyses.

### Alcohol measurement

Alcohol intake was assessed using self-reported drinks per week (DPW), which were derived from participant questionnaires in MVP and UKB, and additionally the AUDIT-C clinical screening tool in MVP. The AUDIT-C is a widely used 3-item version of the Alcohol Use Disorders Identification Test (AUDIT) that screens for hazardous drinking patterns. The AUDIT-C score ranges from 0 to 12, with the items assessing the frequency of alcohol consumption, the number of standard drinks consumed on a typical drinking day, and the frequency of binge drinking (more than 6 drinks on one occasion). In the MVP, DPW was calculated by multiplying the midpoints of drinking frequency and the number of drinks consumed per day. One standard drink is defined as approximately ~14 grams of ethanol. In UKB, participants reported their average weekly or monthly alcohol intake in glasses, which were converted to DPW. To limit potential reverse causation, the earliest recorded alcohol intake was prioritized for analyses, whenever possible, except where explicitly testing for the impact of exposure measurement timing on association with dementia. For the latter, we compared alcohol use as assessed at enrolment surveys. For clinical identification, the AUDIT-C scores can be categorized into three risk groups: non or occasional drinker (0,1), low risk (2-3), high risk (>4).^26,27^ Never and former drinkers were distinguished where recorded. Alcohol use disorder cases were identified using diagnostic codes in the linked EHR.

For the genetic analyses, we included both quantity-frequency traits and problematic alcohol use (PAU)-related traits, which capture somewhat different underlying genetic risk and biology.^15^ For example, PAU, but not AUDIT-C (a quantity-frequency trait) is genetically correlated with multiple psychiatric disorders. ^16,17^ PAU was defined through a meta-analysis that combined data from alcohol use disorder and the AUDIT-P (Alcohol Use Disorders Identification Test – Problem Consumption).^16^ AUDIT-P is a subset of the AUDIT tool that focuses on alcohol-related problems and behaviours.

### Outcome measurement

Given the uncertainty regarding whether specific dementia subtypes are differentially impacted by alcohol consumption, our primary outcome was all-cause dementia. Dementia cases were identified using EHR data and ICD codes (Supplementary Table 19). To minimize the risk of reverse causation, prevalent cases (those diagnosed with dementia at the time of enrollment) were excluded from observational analyses. However, for genetic analyses, both incident (new cases) and prevalent cases were included, as reverse causation is less of a concern in this context.

### Covariates

Potential confounders were identified based on the existing literature. Baseline information on demographic factors, llifestyle behaviors, physical and psychiatric health was collected using self-administered questionnaires. Educational qualifications and household income were treated as categorical variables. Smoking was classified as daily, occasionally, or non-smoker. Body mass index was calculated using self-reported height and weight at enrollment. A history of head injury and post-traumatic stress disorder were recorded as binary variables. Substance use disorders were defined by a lifetime history of opioid or cannabis dependence as indicated by ICD codes in the EHR. Diabetes mellitus was recorded at enrollment survey or in EHR. Mean systolic and diastolic blood pressure was calculated across multiple measurements recorded in the electronic health record. Higher order age terms (age^2^ and age^3^) and age-by-sex interactions were included in the models to account for the non-linear, exponential increase in dementia risk with age and to allow for potential sex-specific age effects.

### Genotyping in MVP

Genotyping and imputation of Million Veteran Program participants has been described previously.^13^ We used genetic data release 4. Briefly, a customized Affymetrix Axiom Array was used for genotyping. Genotype data for biallelic single nucleotide polymorphisms were imputed using Minimac4 and a reference panel from the African Genome Resources panel by the Sanger Institute. Indels and complex variants were imputed independently using the 1000 Genomes (1KG) phase 3 panel and merged in an approach similar to that employed by the UK Biobank. Designation of broad ancestries was based on genetic assignment with comparison to 1KG reference panels.

### Genetic variants

Genetic variants associated with three alcohol use phenotypes - DPW, PAU and AUD – were selected as instrumental variables from the largest available GWAS studies with genome-wide significance (p<5×10^-8^) (Supplementary Tables 7-10 & 18). Instruments were chosen, where possible, from trans-ancestry analyses, but ancestry-specific betas and standard errors were used. As a result, some instruments had higher p values and lower F statistics. Post hoc choice of instruments, genetic models or data based on measured F-statistics can introduce bias. Notably, the widely cited rule that F > 10 avoids bias in instrumental variable analysis is misleading.^28^ We included multi-allelic instruments, as all datasets clearly reported multiple alleles, facilitating comparison across studies. The same covariate set (age, sex, and ancestry principal components) was applied for adjustment in all contributing GWAS. Where available variants were limited, less stringent p value thresholds (p<5×10^-5^) were used, and variants identified in separate ancestry groups. Ancestry-specific linkage disequilibrium clumping was conducted using PLINK v2.0, referencing the 1000 Genomes Project phase 3 LD reference panels, with lead variants identified within a 10,000 kb window and LD r^2^=0.001.

Dementia phenotype definition determines interpretation of Mendelian randomization estimates.^29^ We used genetic associations with all-cause dementia calculated de novo in our primary genetic analyses, given uncertainty about which dementia subtypes alcohol impacts. When outcome data were unavailable, we sought proxies.

### Statistical analyses

Analyses were performed in R (v4.1.2), unless otherwise stated.

#### Observational associations between alcohol and dementia

To estimate the associations between alcohol and incident dementia, Cox proportional hazards models were used. The time at risk was calculated from baseline (when covariates were measured) to either the data of dementia diagnosis or censoring. Influential observations were identified by plotting deviance residuals. The proportional hazards assumption was visually assessed using Schoenfeld residuals and formally with time interactions. For covariates not of primary interest (e.g. age and body mass index), which violated the proportional hazards assumption, stratified models were fitted without the constraint of non-proportionality. Separate baseline hazard functions were fitted for each stratum. The Aalen-Johansen estimator^30^ was used to assess if death was a competing risk. Competing risk of death was accounted for using subdistribution method.^31^ The reference group for alcohol intake was light drinkers, as some current nondrinkers had reduced alcohol intake due to health concerns (‘sick quitters’). Random effects meta-analysis was performed to generate pooled effect sizes across MVP and UKB cohorts (with comparable ancestries).

#### Longitudinal trajectories of alcohol preceding dementia

In MVP, participants had multiple available AUDIT-C scores, which allowed for the examination of changes in drinking behaviors before dementia diagnosis, relevant to reverse causation. Binomial regression models were used, including the following fixed effects: time (from diagnosis/study-end to date of alcohol measurement), dementia status (case/control), enrollment age, sex, education and income, a two-way interaction term between time and dementia status, and a three-way interaction term between time, dementia status and AUDIT-C category (non-drinker, low-risk, high-risk). Wald tests were conducted to estimate the overall effect of interactions between dementia status and time. These tests evaluated whether AUDIT-C trajectory differed by dementia diagnosis. Random effects for participant identification were included. The resulting models were visualized with graphs showing predicted longitudinal trends in AUDIT-C scores for a typical participant.

#### Genome-wide association study of all-cause dementia

Dementia cases were defined by the presence of a relevant ICD 9/10 code in their linked electronic health records. Controls were individuals without such codes. Related individuals (>0.088 Kinship coefficient) were excluded. Ancestry-specific logistic regression was performed in PLINK 2.0 with adjustment for the first 10 principal components, sex, and age. To determine ancestry groupings, the Euclidean distances between each Million Veteran Program participant and the five reference ancestral groups from 1000 Genomes Project were calculated using the first 10 principal components. Participants were assigned to the nearest reference ancestry. A second round of principal component analysis within each assigned ancestral group was performed and outliers with principal component scores >6 standard deviations from the mean of any of the 10 principal components were removed. Variants with call missingness greater than 20% were excluded, as were alleles with minor allele frequency <0.1% in both European and African defined ancestries. After excluding those with missing data, the final sample included 25,473 cases and 425,844 controls for European ancestry, and 5,706 cases and 108,532 controls of African ancestry. The Latin American ancestry group did not have sufficient power for analysis. Heritability for common variants mapped to HapMap3 was calculated using linkage disequilibrium score regression. Additional, genetic correlation between alcohol and dementia phenotypes was estimated.

#### Mendelian randomization

Both linear and nonlinear MR analyses were conducted. The primary analyses used the inverse variance weighted (IVW) method, with several robust methods employed as sensitivity analyses. Reverse Mendelian randomization was conducted to estimate the effects of dementia on alcohol use. Additionally, multivariable MR was performed to assess whether the causal effects of alcohol on dementia changed after adjusting for key potential confounds or mediators.

For nonlinear analysis, five exposure strata were created using individual-level data from European participants in MVP (n=313,873, including 16,932 dementia cases), which provided greater power than UKB for nonlinear analyses. A weighted genetic risk score for alcoholic drinks per week was calculated for each individual by multiplying the number of alcohol-increasing alleles the individual carries by the effect size of the allele with alcohol, and summing across the 641 SNPs. To estimate the nonlinear relationship between genetically-predicted alcohol and dementia, a fractional polynomial method was applied. The sample was divided into five strata using the doubly-ranked method.^32^ The number of strata was chosen to balance assessment of the relationship shape and statistical power. Localized average causal effects (LACE) for each stratum were computed as the ratio of coefficients: the association of the genetic score with the dementia divided by the association of the genetic score with alcohol. These associations were adjusted for age, age^2^, sex and top 10 principal ancestry components. Meta-regression of LACE estimates against the mean of alcohol in each stratum was performed using a flexible semiparametric framework. Nonlinearity was tested by comparing the nonlinear model to a linear model and assessing the trend in LACE estimates. All statistical analyses were conducted using the SUMnlmr package. Negative control analyses of age and sex were performed to rule out bias from confounding.^17^

## Results

### Observational analyses

#### Population characteristics

Across the two cohorts, 559,559 participants were included in observational analyses (Fig 1), of whom 10,564 developed incident all-cause dementia over follow-up in MVP (mean=4.3 years, median=4.4 years, maximum=9.0 years), and 3976 in UKB (mean=12.4 years, median=12.7 years, maximum=15.0 years), consistent with a lower mean age in UKB (Table 1). 28,738 died during follow-up in MVP and 19,296 in UKB. Over 90% of participants in both cohorts reported consuming alcohol at first measurement. Compared to drinkers, current nondrinker groups were older, with a higher proportion of females and had lower educational qualifications. Similar patterns were observed in African (AFR) and Latin American (AMR) ancestry groups (Supplementary Table 1).

#### Observational associations between alcohol use and dementia risk

**Fig 2:**
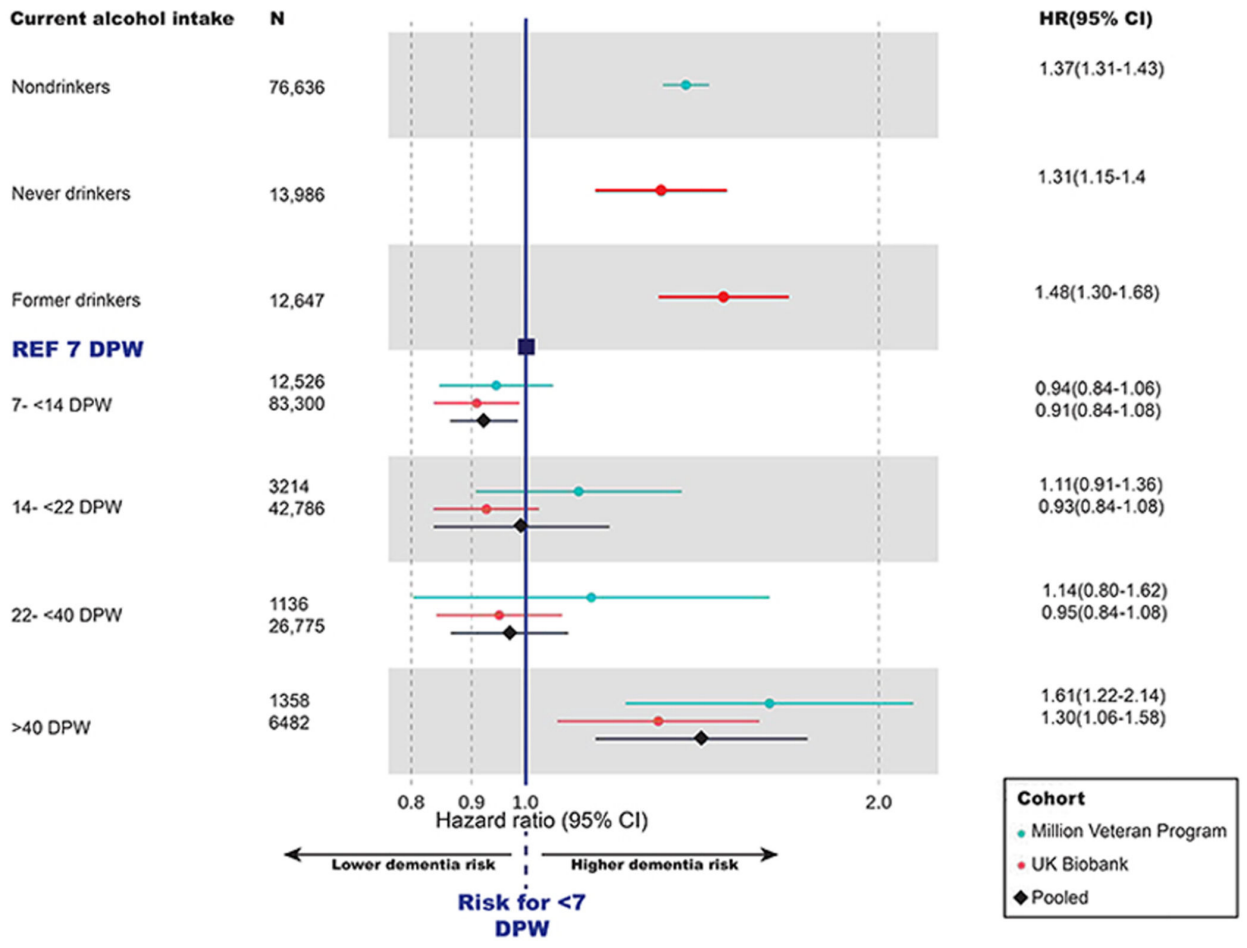
Observational Associations Between Alcohol Intake and Incident Dementia. Hazard ratios (dots or diamonds) and 95% confidence intervals (lines) of all-cause incident dementia according to current alcohol intake, as compared to reference group (solid black line) of individuals consuming <7 drinks per week. Choice of light drinkers as a reference group is motivated by concerns about current-nondrinkers including individuals who previously drank heavily but have reduced their alcohol intake in response to a health concern. Dementia cases were identified by relevant codes in the electronic health record (Supplementary Table 19). Alcohol intake was ascertained at first recording, from the AUDIT-C (Alcohol Use Disorders Identification Test) screening questionnaire in Million Veteran Program, and the baseline questionnaire in UK Biobank. Individuals classed as current nondrinkers in Million Veteran Program were those with an AUDIT-C score of zero. Never and former drinkers could not be distinguished within this group based on the AUDIT-C questions, whereas in UK Biobank never and former drinkers were identifiable from questionnaire answers. Estimates were generated from Cox proportional hazards models, using unrelated European individuals, and adjusted for age, sex, income, education, smoking, body mass index; and additionally in the Million Veteran Program: head injury, post-traumatic stress disorder and substance use. Estimates for other ancestry groups are given in Supplementary Table 4. Pooled estimates across the two cohorts (black diamonds) were generated using random effects meta-analysis.

Conventional observational analyses found a U-shaped relationship between self-reported alcohol intake and incident dementia in both MVP and UKB participants of European (EUR) ancestry (Fig 2) – that is, lowest dementia risk in low-moderate drinkers, rather than nondrinkers. Nondrinkers (irrespective of subdivision into never and former drinkers), heavy (>40 DPW) and dependent drinkers (HR=1.51[1.42-1.60]) had higher incidence of all-cause dementia compared with light drinkers (<7 DPW). In UKB, but not MVP, moderate drinkers (7-14 DPW) had a significantly lower dementia incidence than light drinkers. Accounting for the competing risk of death had little impact on the associations (Supplementary Tables 2 & 3).

A key strength of the MVP cohort is its ancestral diversity, allowing analyses of non-European ancestry populations, previously neglected in alcohol research. Individuals with a history of alcohol use disorder (AUD) had elevated dementia incidence across both AFR (HR=1.44[1.25-1.67]) and AMR 1.58[1.24-2.01]) ancestries (Supplementary Table 4).

#### Alcohol drinking trajectories prior to dementia diagnosis

**Extended Data Figure 1:**
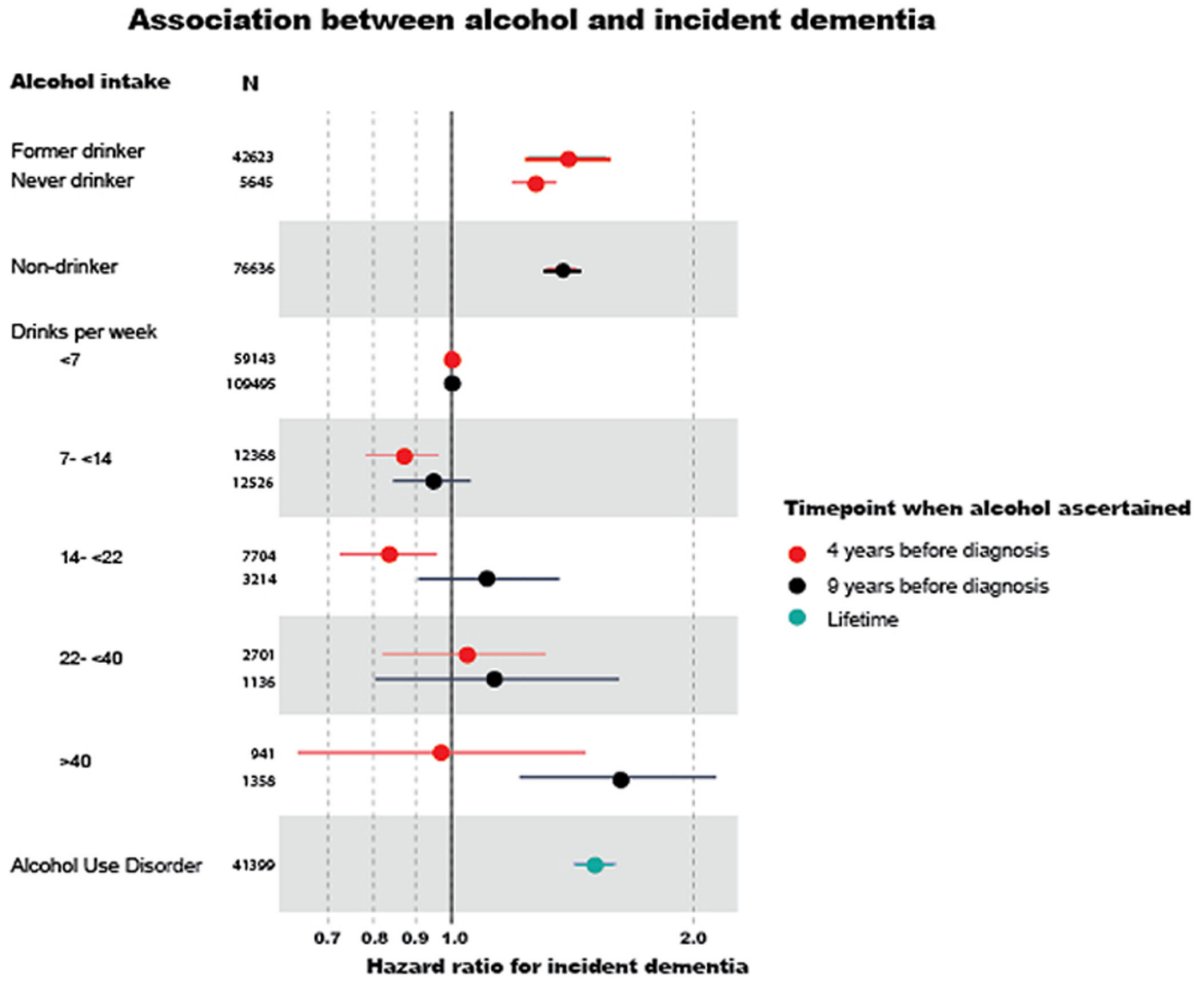
Association of alcohol intake and dementia, according to timing of alcohol self-report, in the Million Veteran Program. Estimates were generated from Cox proportional hazards models in European ancestry individuals from Million Veteran Program, adjusted for: age, sex, income, education, smoking, body mass index, head injury, post-traumatic stress disorder, substance use. The reference group was <7 drinks per week or controls. Alcohol was ascertained on average 9 years before diagnosis from the electronic health record AUDIT-C score, and on average 4 years before diagnosis at baseline surveys. Non-drinkers 9 years before diagnosis were those with AUDIT-C=0 and could not be separated into never and former drinkers.

**Fig 3:**
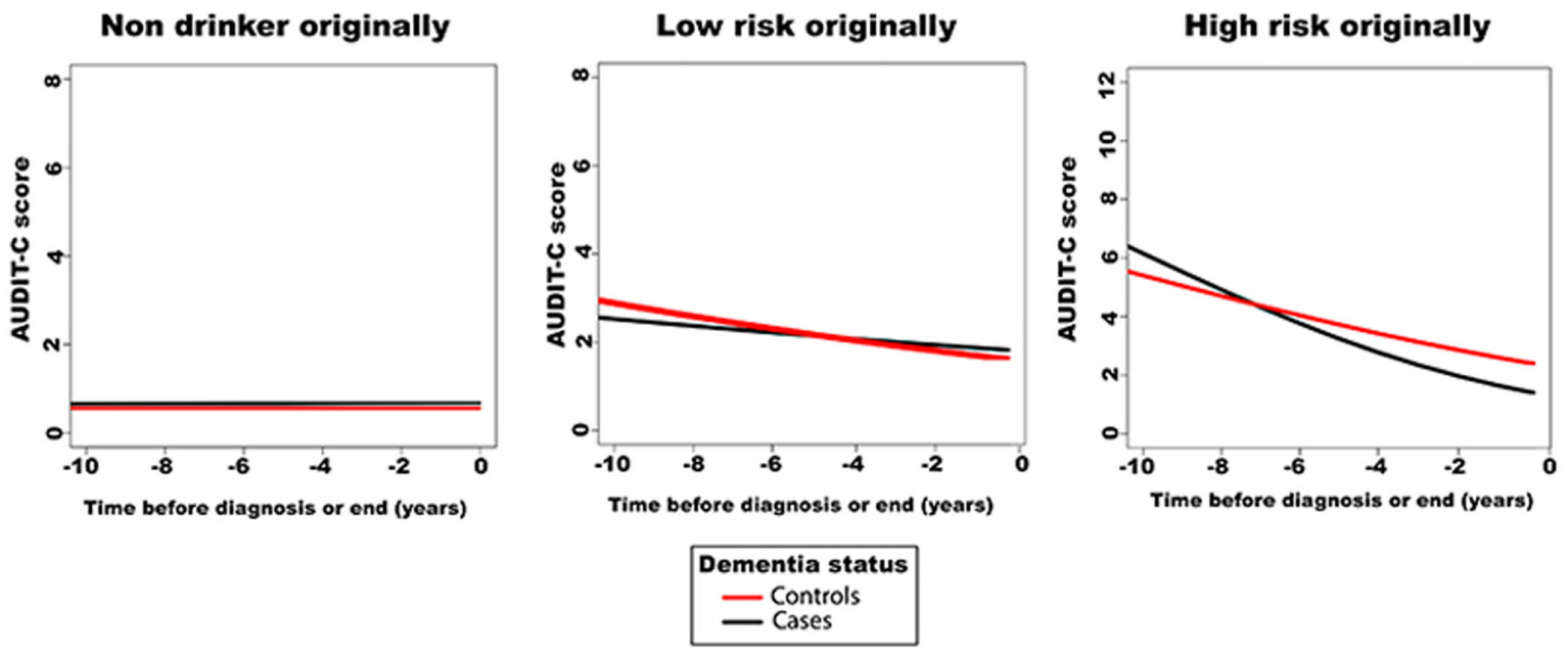
Longitudinal Trends in Alcohol Use Preceding Dementia Diagnosis, in Million Veteran Program. Alcohol use is defined using the AUDIT-C screening questionnaire which was administered and documented at multiple time points in the electronic health record. Dementia status was ascertained by the presence of a relevant clinical code in the electronic health record. The plots show predictions of how drinking behavior changes over time for individuals who develop dementia (cases) and those who remain dementia-free (controls) for: A, individuals who were non- or occasional drinkers at first record (AUDIT-C<1, N=108,544); B, individuals who were low-risk drinkers at first record (AUDIT-C 2- <4, N=51,443); C, individuals who were high-risk drinkers at first record (AUDIT-C >4, N=16,881). Time 0 is the time of diagnosis for cases or last follow up for controls. Predictions are based on mixed effects models adjusted for age, sex, body mass index, smoking, educational qualification and household income. Graphs show predictions for a male participant of average age, body mass index, education and income.

Observational associations between alcohol intake and dementia could be explained by a causal impact of alcohol – alcohol causing dementia - or changes in drinking pattern in prodromal disease – altered, generally decreased, alcohol intake as part of the dementia prodrome (reverse causation). To explore this, we leveraged the longitudinal EHR in MVP to explore how alcohol consumption behaviors (using the Alcohol Use Disorders Identification Test (AUDIT-C)^18^) changed preceding diagnosis of dementia. Nondrinkers were consistent in their abstinence (Fig 3a). Among all drinkers, consumption declined over time. However, this decline was faster among those who went on to develop dementia than for controls (dementia*time beta=0.05, se=0.02, p=0.0003). Furthermore, the accelerated drop in drinking was magnified for those with higher historical drinking (first recorded AUDIT-C>4 dementia status*time beta=0.09, se=0.02, p<0.001) (Fig 3c). Relatedly, associations of alcohol with dementia diagnosis varied with the temporal proximity of the two (Extended Data Figure 1). We compared associations with dementia according to when alcohol intake was measured: 1) the first recorded alcohol record (an average of 9 years prior to dementia diagnosis), and 2) at study enrolment (an average of 4 years prior to dementia diagnosis). When alcohol was measured closer to diagnosis, observed harmful risks of heavy drinking were attenuated, and moderate drinking was associated with an apparent protective effect (7-14 DPW HR=0.87[0.78-0.97]).

### Genetic analyses

#### Bi-ancestry genome-wide association study of all-cause dementia

**Extended Data Figure 2:**
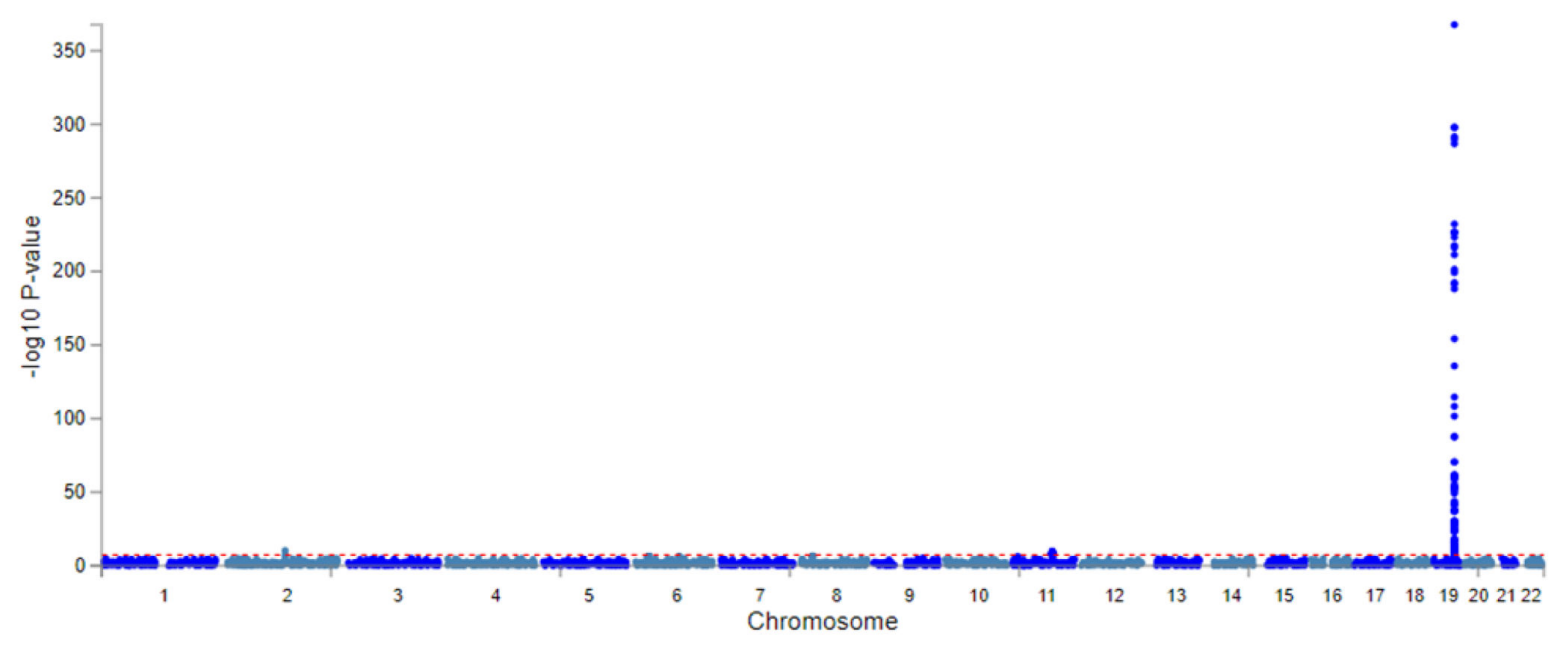
Manhattan plot showing genome-wide associations with all-cause dementia in Million Veteran Program participants of European ancestry. All-cause dementia was determined by the presence of a relevant ICD dementia code in the linked electronic health record (Supplementary Table 19). Analysis was conducted in 25,473 all-cause dementia cases and 425,844 controls of European ancestry who were unrelated.

**Extended Data Figure 3:**
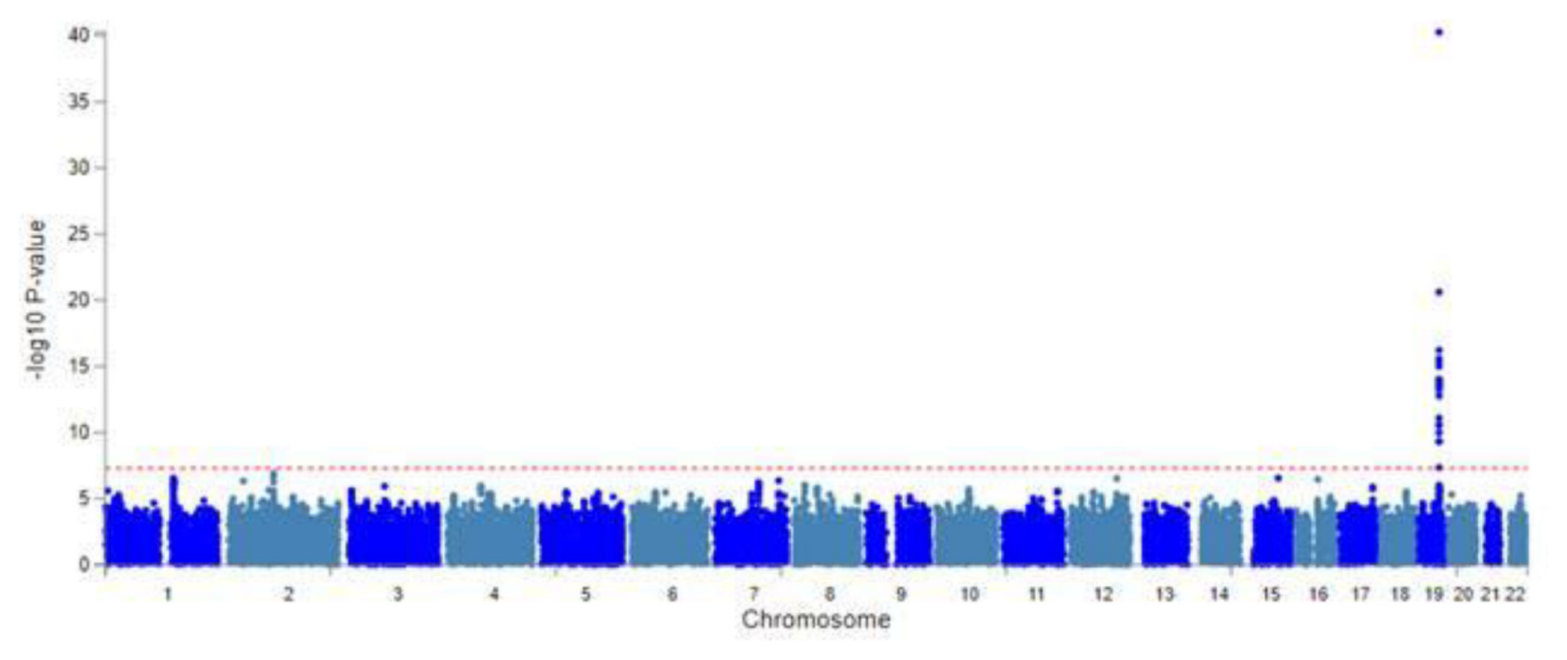
Manhattan plot showing genome-wide associations with all-cause dementia in Million Veteran Program participants of African American ancestry. All-cause dementia was determined by the presence of a relevant ICD dementia code in the linked electronic health record (Supplementary Table 19). Analysis was conducted in 5,706 all-cause dementia cases and 108,532 controls of African ancestry who were unrelated.

To increase power for all-cause dementia in older adults, we conducted a de novo GWAS in MVP to generate necessary data for our analyses. Four independent SNPs (*PICALM, APOE, BCAM/NECTIN2, BIN1/NIFKP9*) were associated with all-cause dementia at genome-wide significance (p<5×10^-8^) in EUR (Supplementary Table 5 & Extended Data Figure 2 and one SNP (*APOE*) in AFR (Extended Data Figure 3). The strongest association in EUR was with rs429358 (p=1.14×10^-314^), in the *APOE* gene. All-cause dementia heritability was h^2^=0.01 on the liability scale. There were significant genetic correlations between three examined alcohol phenotypes and all-cause dementia (Supplementary Table 6). These were strongest for AUD (R_g_=0.45[0.10-0.80]) and weakest for DPW (R_g_=0.19[0.01-0.36]).

#### Potential causal associations between alcohol use and dementia

**Fig 4:**
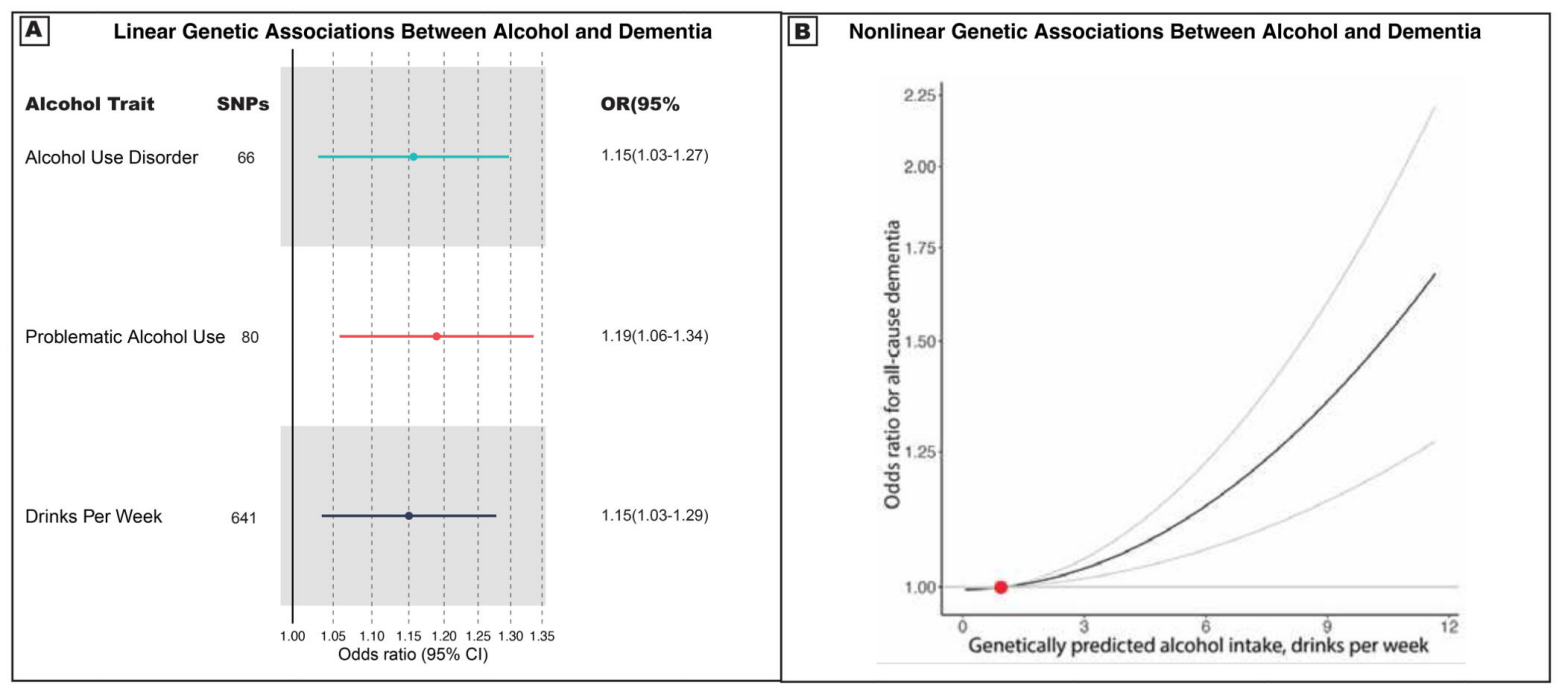
Genetic Associations Between Alcohol Use and Dementia. A) Forest plot shows causal odds ratios for all-cause dementia for either a 1 standard deviation increase in log-drinks per week or a two-fold increase in prevalence of problematic alcohol use of alcohol use disorder. A stringent (p<5×10^-8^) threshold was used to select genetic instruments from genome-wide association analyses. Alcohol use is characterized by three phenotypes: a clinical diagnosis of Alcohol Use Disorder in the electronic health record, problematic use (meta-analyzing Alcohol Use Disorder and AUDIT-P, a screening tool for problematic drinking), and number of drinks per week. All-cause dementia was determined by any clinical dementia diagnosis in the electronic health record. Estimates were generated from one- and two-sample inverse variance weighted Mendelian randomization from a combined sample size across source genome-wide association studies of over 2.4 million individuals (Supplementary Table 18). B) Nonlinear Mendelian randomization was performed using the doubly-ranked method, in unrelated European ancestry Million Veteran Program participants (n=313,873, 16,932 dementia cases). The x-axis shows the alcohol intake in drinks per week at enrollment. The y-axis shows the odds ratio for the respective all-cause dementia risk. The gradient at each point of the curve is the localized average causal effect. Values are based on mean intake in five strata of exposure (quintiles). Grey lines represent the 95% confidence intervals. The reference value (in red) for alcohol intake was taken as 1 drink per week. P-value for linearity: 0.8 (a non-significant result indicates that the best fitting quadratic model is not strongly preferred over the linear model), p-value for trend: 0.5 (a nonsignificant result indicates no strong evidence for trend in the estimates calculated in different strata).

In the linear MR analysis, three alcohol phenotypes were used as exposures: 1) DPW, instrumented using 641 independent genetic variants; 2) PAU, with 80 genetic variants; and 3) AUD, with 66 genetic variants (Supplementary Tables 7-10). Higher genetic liability for all three alcohol use measures was associated with an increased risk of all-cause dementia in European ancestry participants (Fig 4). Specifically, a two-fold increase in the prevalence of AUD was associated with a 16% increase in dementia risk (IVW OR=1.16[1.03-1.30]).^19^ Additionally, a one standard deviation increase in log-transformed DPW (corresponding to an increase from 1 to 3 DPW, or 5 to 16 DPW) was associated with a 15% increased risk of dementia risk (IVW OR=1.15[1.03-1.27]). To assess the strength of causal evidence, we conducted several sensitivity analyses (Supplementary Table 11-14). These included: correcting for sample overlap bias using MRlap, removing outliers using MR-PRESSO, and testing for reverse causality with reverse MR, which did not undermine the key findings. In contrast, some heterogeneity across SNP estimates and pleiotropy (for DPW) were observed. We also used multivariable MR to control for genetic correlations with socioeconomic factors and smoking. After adjusting for post-traumatic stress disorder, associations between alcohol use disorder and dementia were attenuated. However, these associations remained significant when controlling for smoking or cannabis use. Associations among African ancestry were weaker, likely due to lower statistical power of analyses (Supplementary Table 11).

Next, we conducted nonlinear MR analyses to examine the shape of the relationship between alcoholic consumption (measured as drinks per week) and dementia which cannot be examined using linear MR. Nonlinear MR necessitates individual level genetic data and was performed in MVP, which had a large number of dementia cases and thus power to detect effects. Unlike in the observational analyses, no U-shaped association was found between alcohol intake and dementia, and no protective effects of low levels of alcohol intake were observed (Fig 4 & Supplementary Table 15). Instead, dementia risk increased consistently across the entire range of genetically predicted DPW. For a subgroup (stratum) of the population with a mean consumption of 12 drinks per week, we found evidence for a causal relationship with dementia (OR=1.09[1.04 to 1.15)]) (Supplementary Table 15). To validate the genetic instruments used for alcohol, we employed age and sex as negative controls,^20^ which should not be causally related to alcohol consumption. No significant associations were found between genetically-predicted alcohol consumption and age or sex across different strata, supporting the conclusion that the nonlinear MR estimates reflect causal effects rather than confounding (Supplementary Tables 16 & 17).

## Discussion

Conventional observational analyses showed a U-shaped association between alcohol consumption and dementia (as has been seen in previous studies) seeming to support that low or moderate alcohol use is associated to lower dementia risk than no alcohol at all. However, less-confounded genetic analyses provided evidence for a monotonically increasing causal risk of alcohol consumption on all-cause dementia, contradicting the interpretation that one can drink alcohol to decrease dementia risk. Furthermore, drinkers who went on to develop dementia typically reduced their alcohol consumption in the years preceding diagnosis, suggesting that apparent protective effects of moderate drinking may be a consequence of reverse causation.

The reduction in alcohol use prior to dementia onset aligns with findings from studies of other putative dementia risk factors, including body mass index.^21^ This has important general implications for study design and for the interpretation of prior studies. Reverse causation is further supported by our finding of higher incidence of dementia amongst nondrinkers, in line with previous studies.^5^ These groups may include ‘sick quitters’—individuals with prior heavy use^13^ —often with earlier deaths, which may explain their higher dementia risk. Additionally, nondrinkers tended to have lower socioeconomic status and education levels, both of which are associated with poorer pre-morbid cognitive function and increased vulnerability to dementia. Imperfect control means that even though these factors were accounted for statistically, measurement errors mean residual confounding could still underlie apparent observational associations with dementia. In line with many,^1,4^ but not all,^5^ prior studies, heavy alcohol use associated with an increased dementia risk. Variability in alcohol phenotypes, timing of alcohol self-report (especially between mid-life and late-life),^5^ and differing methods for adjusting for confounding factors likely contribute to these discrepancies. Ethnic diversity in alcohol use and dementia risk has been understudied, but our analyses across European, African and Latin American ancestry populations observed similar risks associated with alcohol use disorder.^5^

Importantly, MR, which has a lower risk of both residual confounding and reverse causation, supported a causal role of alcohol use, drinks per week, problematic and dependent use, in increasing dementia risk. We suggest two explanations for discrepancies between our MR results and previous null result studies.^14^ Earlier studies considered a narrow range of alcohol phenotypes and focused on late-onset Alzheimer’s disease, with one exception,^22^ whereas we included a broader range of alcohol and neurodegenerative pathologies. Second, our study had greater statistical power. Furthermore, our nonlinear analyses did not support the previously hypothesized protective effects of low alcohol consumption on dementia, and instead showed a monotonically increasing risk with alcohol dose. These results are consistent with a lower powered one-sample MR study in current drinks of White British ancestry,^12^ as well as neuroimaging findings^.3,23,24^ This finding has key public health implications, as it challenges the long-standing notion that moderate alcohol intake might have a protective effect on the brain. Several important sensitivity analyses strengthened our findings, including use of age and sex as negative controls to rule out potential biases. Additionally, the consistency of these findings across multiple alcohol phenotypes enhances the robustness of our conclusions.

Despite the strengths of our study – a large sample size, cross-ancestry analyses, and triangulation of observational and genetically informed approaches – there are limitations. Most notably, analyses had greatest power to detect effects in EUR groups, and dementia diagnoses from EHR may be subject to ascertainment bias, though this would likely bias associations toward the null. MR methods rely on unverifiable assumptions, and the estimates we derived reflect the accumulated effect of alcohol over a lifetime which do not necessarily translate into potential consequences resulting from an adult life intervention. Heterogeneity in estimates between genetic variants may plausibly be due to alcohol acting via different pathways and organs to damage the brain, or failure of the homogeneity or linearity assumptions.^25^ The latter were tested and held. Nonlinear Mendelian randomization estimates at the lower alcohol doses have less precision than those at higher doses, with potential implications for detecting J-shaped relationships, however, negative controls for age and sex did not indicate bias across strata.^20^

In summary, our study shows that alcohol consumption has a causal detrimental effect on dementia risk, with no evidence supporting the previously suggested protective effect of moderate drinking. The pattern of reduced alcohol use before dementia diagnosis observed in our study underscores the complexity of inferring causality from observational data, especially in aging populations. Our findings highlight the importance of considering reverse causation and residual confounding in studies of alcohol and dementia, and they suggest that reducing alcohol consumption may be an important strategy for dementia prevention.

## Extended Data

**Extended Data Figure 4:**
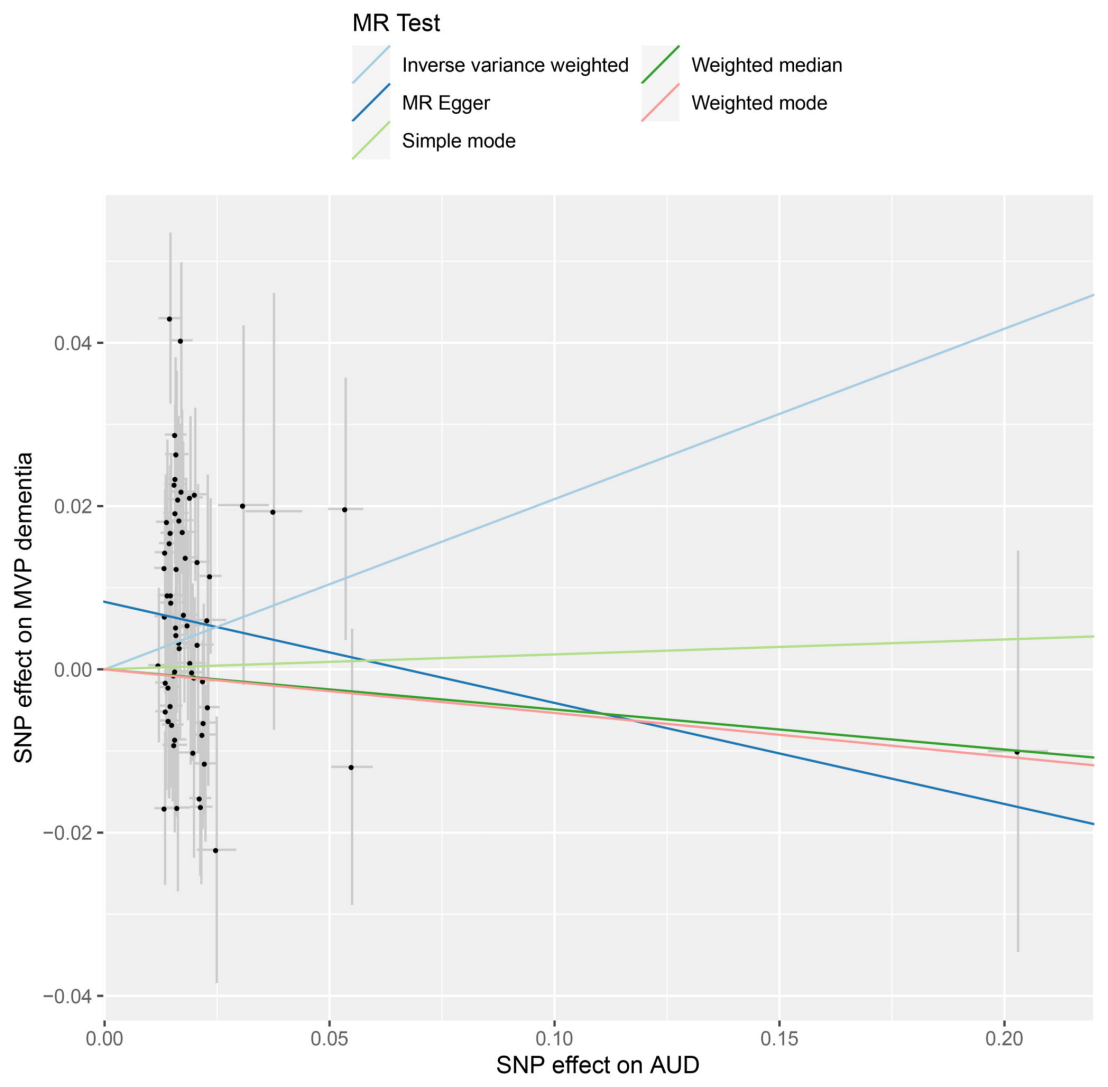
Scatter plot of Mendelian randomization analysis of alcohol use disorder and all-cause dementia. All-cause dementia and alcohol use disorder (AUD) were defined by the presence of a relevant ICD code in the electronic health record (Supplementary Table 19). Genetic associations with dementia were calculated *de novo* in this study in Million Veteran Program, and with alcohol use disorder from Zhou et al.^1^

**Extended Data Figure 5:**
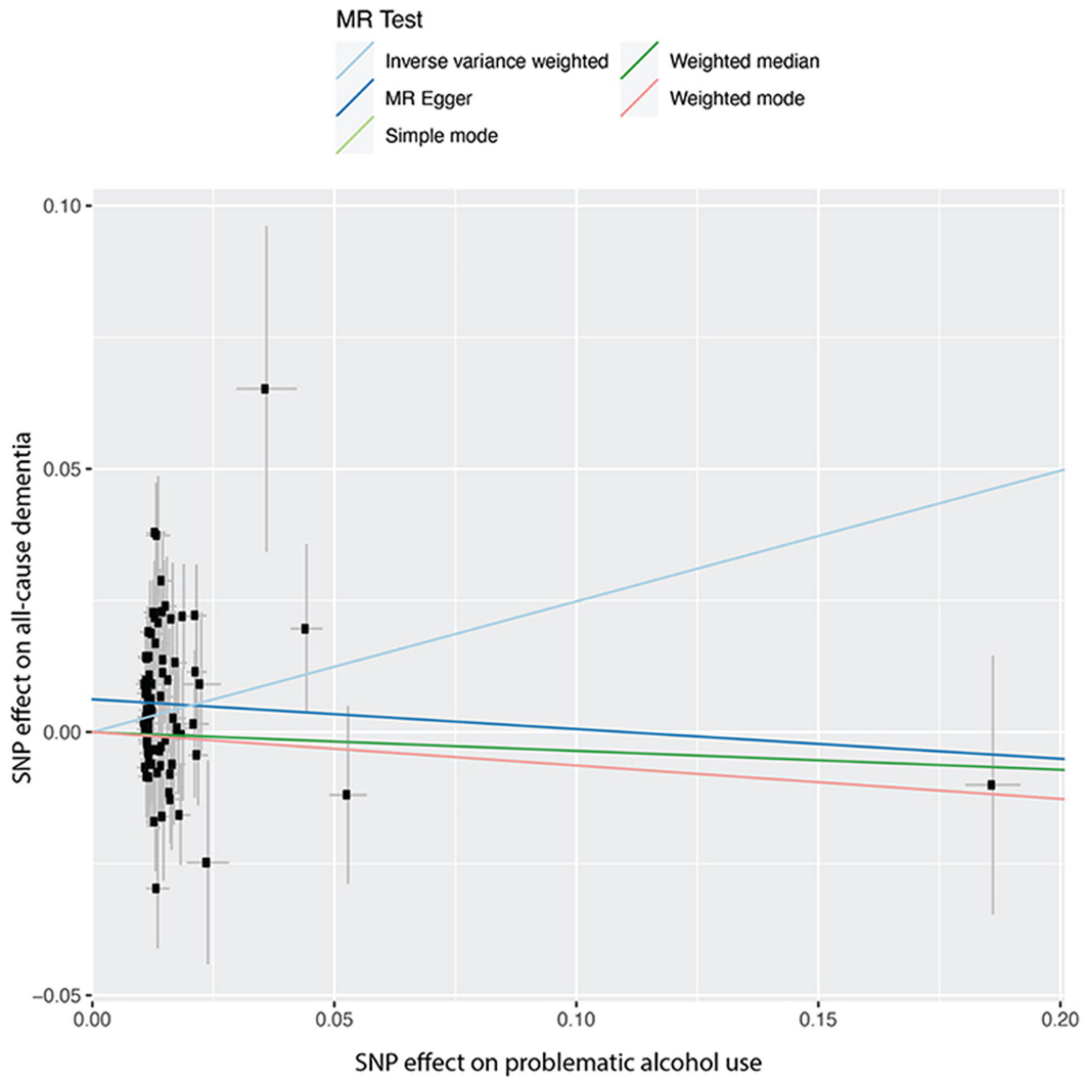
Scatter plot of Mendelian randomization analysis of problematic alcohol use and all-cause dementia. All-cause was defined by the presence of a relevant ICD code in the electronic health record (Supplementary Table 19). Problematic alcohol use was defined by meta-analyzing alcohol use disorder and AUDIT-P. Genetic associations with dementia were calculated *de novo* in this study in Million Veteran Program, and with problematic alcohol use from Zhou et al.^1^

**Extended Data Figure 6:**
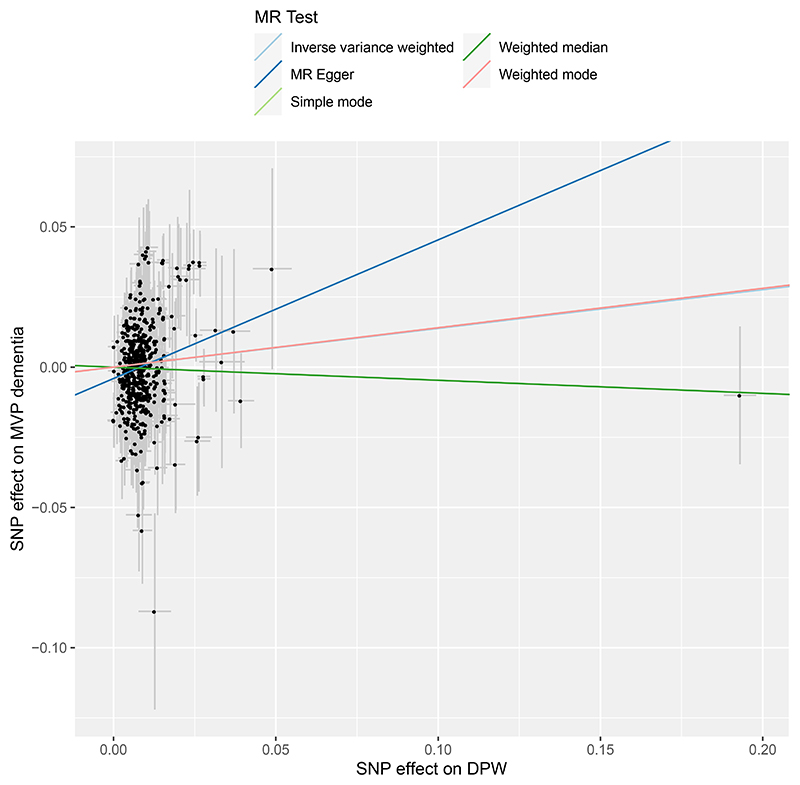
Scatter plot of Mendelian randomization analysis of drinks per week and all-cause dementia. All-cause was defined by the presence of a relevant ICD code in the electronic health record (Supplementary Table 19). Genetic associations with dementia were calculated de novo in this study in Million Veteran Program, and with drinks per week from Saunders et al.^2^

## Supplementary Material

Extended data figures

## Figures and Tables

**Table 1:** Participant characteristics.

			Million Veteran Program							UK Biobank			
Drinking status	*Nondrinker*	*<7* *DPW*	*7- <14* *DPW*	*14-<22* *DPW*	*22-<40* *DPW*	>*40* *DPW*	*Never*	*Former*	*<7* *DPW*	*7- <14* *DPW*	*14-<22* *DPW*	*22-<40* *DPW*	>*40* *DPW*
**No.(%)**	76,636 (37.5)	109,495 (53.6)	12,525 (6.1)	3216 (1.6)	1136 (0.6)	1358 (0.7)	13,986 (4.5)	12,647 (4.0)	126,447 (40.5)	83,300 (13.7)	42,786 (26.7)	26,775 (8.6)	6482 (2.1)
**Age,** mean years (S.D.)	67.5 (10.6)	66.8 (12.1)	64.1 (11.1)	62.8 (10.2)	59.3 (11.4)	57.5 (10.3)	56.7 (8.6)	56.7 (7.9)	56.2 (8.1)	56.1 (8.0)	56.2 (7.9)	56.2 (7.9)	55.7 (7.7)
**Sex,** No.(%)													
Female	4457 (5.8)	6360 (5.8)	322 (2.6)	96 (3.0)	37 (3.3)	47 (3.5)	9742 (69.7)	6685 (52.9)	81,525 (64.5)	38,447 (46.2)	13,241 (30.9)	4788 (17.9)	741 (11.4)
**Smoking status,** No.(%)													
Regular ^b^	12,705 (16.6)	15,875 (14.5)	2961 (2.4)	1139 (3.5)	463 (4.1)	703 (5.2)	881 (6.3)	1886 (14.9)	8978 (7.1)	7657 (9.2)	5593 (13.1)	5101 (19.1)	1924 (29.7)
Never	59,626 (77.8)	85,084 (77.7)	8285 (66.1)	1763 (54.8)	563 (49.6)	511 (37.6)	11,409 (81.6)	5568 (44.0)	79,090 (62.5)	42,488 (51.0)	17,642 (41.2)	8715 (32.5)	1644 (25.4)
**Household income,** No.(%)^c^													
<*$10,000*	4122 (5.4)	3952 (3.6)	683 (5.5)	322 (10.0)	160 (14.1)	270 (19.9)	5839 (41.7)	5191 (41.0)	26,715 (21.1)	13,384 (16.1)	6611 (15.5)	4770 (17.8)	1602 (24.7)
>*$150,100*	789 (1.0)	2864(2.6)	295(2.4)	49(1.5)	6(0.5)	9(0.7)	330 (2.4)	356 (2.8)	6288(5.0)	6121 (7.3)	3610 (8.4)	1991(7.4)	370(5.7)
**Educational qualifications,** No.(%)^d^													
Less than high school	4565 (6.0)	3150 (2.9)	384 (3.1)	163 (5.1)	53 (4.7)	67 (4.9)	3196 (22.9)	2976 (23.5)	16,336 (12.9)	9981 (12.0)	5318 (12.4)	3894 (14.5)	1202 (18.5)
Professional/doctoral degree	1839(2.4)	3720(3.4)	287(2.3)	43(1.3)	11(1.0)	15(1.1)	4071(29.1)	3617(28.6)	46,569(36.8)	32,835(39.4)	16,305(38.1)	8937(33.4)	1811(27.9)
**Mean time at risk,** days(S.D)	4.5(2.3)	4.5(2.3)	4.4(2.3)	4.4(2.2)	4.5(2.2)	4.4(2.2)	12.4(1.9)	12.1(2.3)	12.4(1.6)	12.6(1.7)	12.5(1.8)	12.4(2.0)	12.2(2.4)

Abbreviations: DPW – drinks per week, no. – number, S.D. – standard deviation.

a The demographic data were collected at baseline surveys. Data are shown for unrelated participants of European ancestry. Other ancestry group data are reported in Supplementary Table 1.

b Defined as daily smoker in Million Veteran and current smoker in UK Biobank.

c The equivalent categories of household income in UK Biobank were <£18,000 and >£100,000. Highest and lowest categories only displayed.

d In UK Biobank the lowest educational category was no formal qualifications. Highest and lowest categories only displayed.

e Cases were based on physician diagnosis of any cause dementia, as ascertained by the presence of a relevant ICD dementia code in the electronic health record. The full list of codes is given in Supplementary Table 19.

## Data Availability

Access to UK Biobank data is available following a successful application. Access to Million Veteran Program summary statistics is available through via dbGAP.

## Key messages

### What is already known on this topic

Previous observational studies have reported a J-shaped association between alcohol consumption and dementia, suggesting that light-to-moderate drinking may be protective. However, these findings may reflect residual confounding or reverse causation, rather than a causal effect.

### What this study adds

In the largest combined observational and genetic study to date, light alcohol consumption was associated with the lowest dementia risk observationally, but genetic analyses showed a monotonic increasing dementia risk with increased alcohol intake. Mendelian randomization suggests a causal role of alcohol consumption in increasing dementia risk, with no evidence supporting a protective effect at any consumption level.

### How might this study affect research, practice or policy

These findings challenge the notion that low levels of alcohol are neuroprotective and suggest that public health efforts to reduce alcohol use disorder could significantly lower dementia incidence. Halving the population prevalence of alcohol use disorder may reduce dementia cases by up to 16%, highlighting alcohol reduction as a potential strategy in dementia prevention policies.
