# Supplementary material for: Alcohol Use and Risk of Dementia in Diverse Populations
Source: BMJ Evid Based Med. Author manuscript; Available in PMC 2026 Jan 20. (PMC7618643; doi:10.1136/bmjebm-2025-113913)
Supplement: Extended data figures [file EMS209260-supplement-Extended_data_figures.docx]

Table of Contents

[Extended Data Figure 1: Association of alcohol intake and dementia, according to timing of alcohol self-report, in the Million Veteran Program. 1](#_Toc194929908)

[Extended Data Figure 2: Manhattan plot showing genome-wide associations with all-cause dementia in Million Veteran Program participants of European ancestry. 1](#_Toc194929909)

[Extended Data Figure 3: Manhattan plot showing genome-wide associations with all-cause dementia in Million Veteran Program participants of African American ancestry. 2](#_Toc194929910)

## Extended Data Figure 1: Association of alcohol intake and dementia, according to timing of alcohol self-report, in the Million Veteran Program.


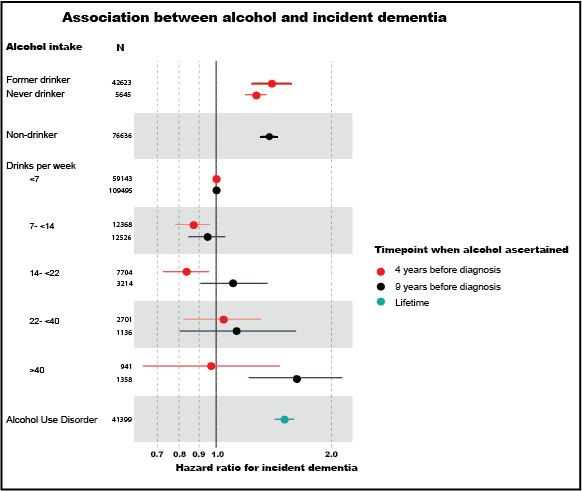


Estimates were generated from Cox proportional hazards models in European ancestry individuals from Million Veteran Program, adjusted for: age, sex, income, education, smoking, body mass index, head injury, post-traumatic stress disorder, substance use. The reference group was <7 drinks per week or controls. Alcohol was ascertained on average 9 years before diagnosis from the electronic health record AUDIT-C score, and on average 4 years before diagnosis at baseline surveys. Non-drinkers 9 years before diagnosis were those with AUDIT-C=0 and could not be separated into never and former drinkers.

##

## Extended Data Figure 2: Manhattan plot showing genome-wide associations with all-cause dementia in Million Veteran Program participants of European ancestry.


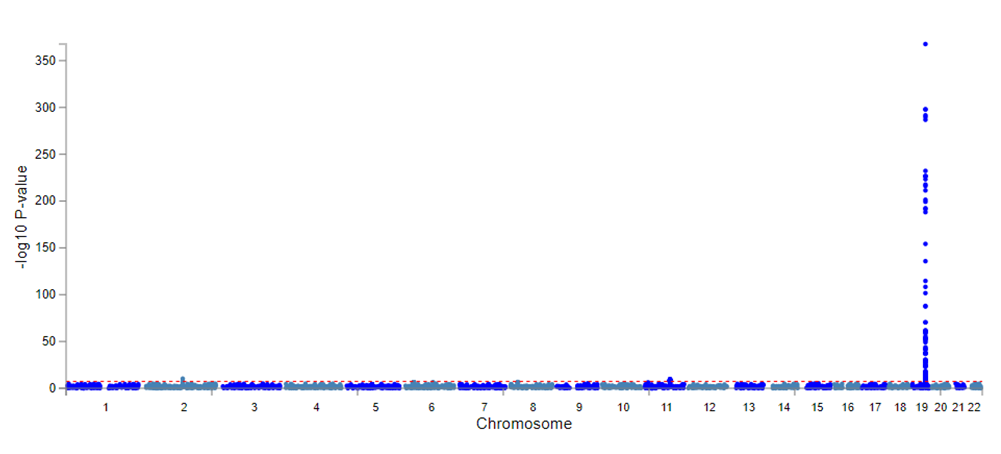


All-cause dementia was determined by the presence of a relevant ICD dementia code in the linked electronic health record (Supplementary Table 19). Analysis was conducted in 25,473 all-cause dementia cases and 425,844 controls of European ancestry who were unrelated.

## Extended Data Figure 3: Manhattan plot showing genome-wide associations with all-cause dementia in Million Veteran Program participants of African American ancestry.

**
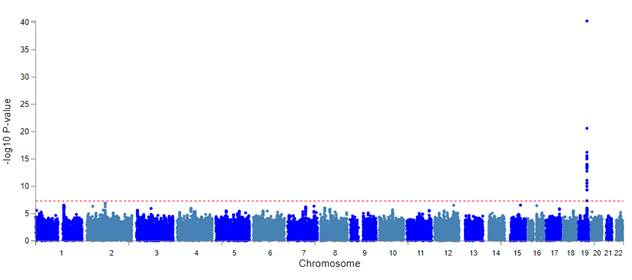
**

All-cause dementia was determined by the presence of a relevant ICD dementia code in the linked electronic health record (Supplementary Table 19). Analysis was conducted in 5,706 all-cause dementia cases and 108,532 controls of African ancestry who were unrelated.

##

## Extended Data Figure 4: Scatter plot of Mendelian randomization analysis of alcohol use disorder and all-cause dementia.

All-cause dementia and alcohol use disorder (AUD) were defined by the presence of a relevant ICD code in the electronic health record (Supplementary Table 19). Genetic associations with dementia were calculated *de novo* in this study in Million Veteran Program, and with alcohol use disorder from Zhou et al.^1^

## Extended Data Figure 5: Scatter plot of Mendelian randomization analysis of problematic alcohol use and all-cause dementia.


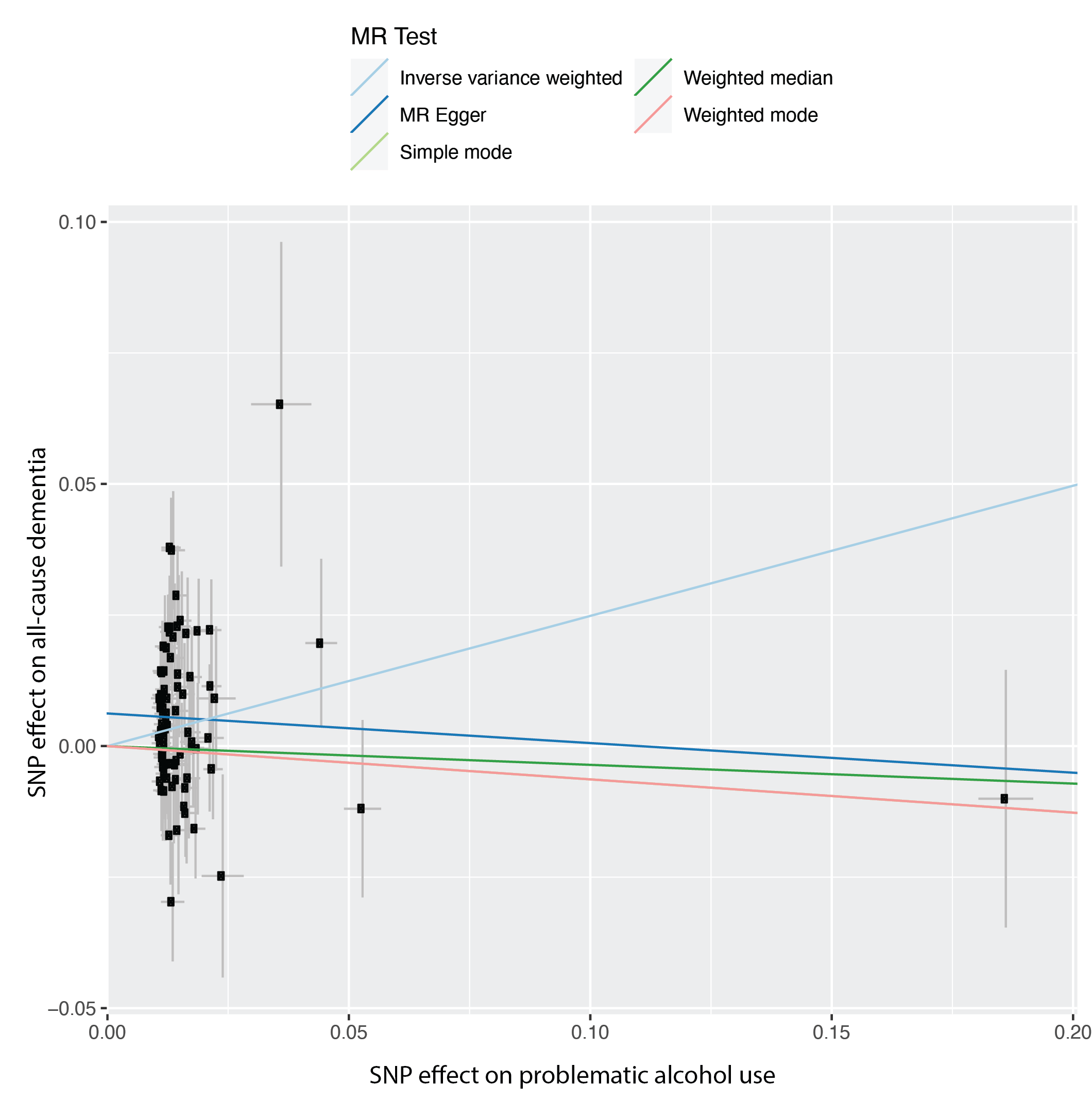


All-cause was defined by the presence of a relevant ICD code in the electronic health record (Supplementary Table 19). Problematic alcohol use was defined by meta-analyzing alcohol use disorder and AUDIT-P. Genetic associations with dementia were calculated *de novo* in this study in Million Veteran Program, and with problematic alcohol use from Zhou et al.^1^

## Extended Data Figure 6: Scatter plot of Mendelian randomization analysis of drinks per week and all-cause dementia.

All-cause was defined by the presence of a relevant ICD code in the electronic health record (Supplementary Table 19). Genetic associations with dementia were calculated de novo in this study in Million Veteran Program, and with drinks per week from Saunders et al.^2^

# References

1. Zhou, H.*, et al.* Multi-ancestry study of the genetics of problematic alcohol use in over 1 million individuals. *Nature Medicine*, 1-9 (2023).

2. Saunders, G.R.*, et al.* Genetic diversity fuels gene discovery for tobacco and alcohol use. *Nature*, 1-7 (2022).
